# Microsatellite and flow cytometry analysis to help understand the origin of *Dioscorea alata* polyploids

**DOI:** 10.1093/aob/mct145

**Published:** 2013-08-01

**Authors:** A. Nemorin, J. David, E. Maledon, E. Nudol, J. Dalon, G. Arnau

**Affiliations:** 1 CIRAD (Centre de Coopération Internationale en Recherche Agronomique pour le Développement), Station de Roujol, 97170 Petit Bourg, Guadeloupe, France; 2 UMR AGAP, Montpellier Supagro, 2, place Viala, 34060 Montpellier Cedex 2, France

**Keywords:** *Dioscorea alata*, endosperm balance, 2*n* gametes, bilateral sexual polyploidization, triploid origin, polyploidy

## Abstract

**Background and Aims Dioscorea alata:**

is a polyploid species with a ploidy level ranging from diploid (2*n* = 2*x* = 40) to tetraploid (2*n* = 4*x* = 80). Ploidy increase is correlated with better agronomic performance. The lack of knowledge about the origin of *D. alata* spontaneous polyploids (triploids and tetraploids) limits the efficiency of polyploid breeding. The objective of the present study was to use flow cytometry and microsatellite markers to understand the origin of *D. alata* polyploids.

**Methods:**

Different progeny generated by intracytotype crosses (2*x* × 2*x*) and intercytotype crosses (2*x* × 4*x* and 3*x* × 2*x*) were analysed in order to understand endosperm incompatibility phenomena and gamete origins via the heterozygosity rate transmitted to progeny.

**Results:**

This work shows that in a 2*x* × 2*x* cross, triploids with viable seeds are obtained only via a phenomenon of diploid female non-gametic reduction. The study of the transmission of heterozygosity made it possible to exclude polyspermy and polyembryony as the mechanisms at the origin of triploids. The fact that no seedlings were obtained by a 3*x* × 2*x* cross made it possible to confirm the sterility of triploid females. Flow cytometry analyses carried out on the endosperm of seeds resulting from 2*x* × 4*x* crosses revealed endosperm incompatibility phenomena.

**Conclusions:**

The major conclusion is that the polyploids of *D. alata* would have appeared through the formation of unreduced gametes. The triploid pool would have been built and diversified through the formation of 2*n* gametes in diploid females as the result of the non-viability of seeds resulting from the formation of 2*n* sperm and of the non-viability of intercytotype crosses. The tetraploids would have appeared through bilateral sexual polyploidization via the union of two unreduced gametes due to the sterility of triploids.

## INTRODUCTION

*Dioscorea alata* is a monocot that belongs to the family Dioscoreaceae. This gender includes >600 species (Ayensu and Coursey, 1972) of which the three main cultivated species are *D. rotundata*, *D. alata* and *D. trifida*. Yams are an important food crop in tropical and sub-tropical regions. They are dioecious herbaceous vines cultivated for their starchy tubers. They are exclusively propagated by vegetative multiplication by means of small tubers or small pieces of tubers. New combinations can be obtained via sexual reproduction, and breeding new cultivars has proven to be an efficient method for genetic improvement (Abraham and Nair, 1991; Egesi and Asiedu, 2002; Arnau *et al.*, 2010, 2011).

*Dioscorea alata* is a polyploid species with diploid (2*n* = 40), triploid (2*n* = 60) and tetraploid (2*n* = 80) cytotypes (Arnau *et al.*, 2009). The origin of this species is still a matter of debate because it has not yet been clearly identified in its wild state in nature, whereas wild forms have been mentioned by Shajeela *et al.* (2011). Ploidy increase is correlated with growth vigour, higher and more stable tuber yield and increased tolerance to abiotic and biotic stress (Malapa *et al.*, 2005; Lebot, 2009; Arnau *et al.*, 2010). Diversity studies have shown that the most common forms are diploids, followed by triploids, and that tetraploids are rare and only exist in diversification centres in Asia and the South Pacific (Abraham and Nair, 1991; Arnau *et al.*, 2009; Lebot, 2009). Recent studies have demonstrated a tetrasomic segregation for *D. alata* tetraploid clones (2*n* = 4*x* = 80) (Nemorin *et al.*, 2012). Autotetraploidy confers several advantages, of which hybrid vigour, also known as heterosis, is among the most common (Gallais, 2003; Parisod *et al.*, 2010). Autotetraploids can be formed from diploids in a variety of ways (Harlan and de Wet, 1975; Ramsey and Schemske, 1998), one of which is thought to involve the combination of two unreduced (2*n*) gametes (bilateral sexual polyploidisation, BSP) (Bretagnolle and Thompson, 1995; Ramsey and Schemske, 1998). There are two major ways to produce 2*n* gametes: by first division restitution (FDR) or by second division restitution (SDR) (Mok and Peloquin, 1975). These two cytological events have different genetic consequences. In fact, FDR 2*n* gametes transmit the major part of parental heterozygosity to progeny, whereas SDR 2*n* gametes are rather homozygous. Because BSP would only occur with the joint probability of two events of low likelihood, it is considered quite rare in natural populations (Husband, 2004). However, tetraploids have been obtained by BSP in several species such as *Dactylis glomerata* (Bretagnolle and Lumaret, 1995), *Trifolium pratense* (Parrott and al., 1985) and diploid relatives of *Solanum tuberosum* (Mendiburu and Peloquin, 1977; Hutten *et al.*, 1995).

Alternatively, tetraploids may be produced in two steps via a triploid intermediary, through a process known as the triploid bridge (unilateral sexual polyploidization, USP). Tetraploids have been obtained by USP in several species such as *Cucumis sativus* (Diao *et al.*, 2009), *Achillea borealis* (Ramsey, 2007) and *Chamerion angustifolium* (Husband, 2004). Triploids that are formed through the union of a haploid (*x*) and a diploid unreduced (2*x*) gamete are of varying fertility depending on the species (Ortiz and Vuylsteke, 1995; Burton and Husband, 2001; Park *et al.*, 2002). Meiosis of triploids is irregular and results in a majority of aneuploid gametes. Euploid gametes that are haploid, diploid or triploid would be formed at the expected frequencies of 1/2^*x*^, 1/2^*x*^ + 1/2^*x*^, 1/2^*x*^ + 1/2^*x*^ + 1/2^*x*^, respectively (Lange and Wagenvoort, 1973), where *x* is the basic chromosome number. Depending on their fertility and the ploidy of their functional gametes (e.g. *n* = *x*, 2*x* or 3*x*), triploids may produce tetraploids through the union of a balanced triploid gamete (3*x*) with a haploid gamete (*x*) of a diploid or a triploid. Non-gametic reduction in triploids can increase the frequency of balanced gametes (2*x* and 3*x*) by SDR and FDR, respectively, as is the case in *C. angustifolium* (Husband, 2004).

Unions of gametes of different ploidy levels are expected to interfere with seed development. In angiosperms, seeds are obtained by double fertilization (fertilization of the oosphere, producing the embryo, and fertilization of the central cell, leading to the formation of the endosperm). Because the central cell of most flowering plant species is homodiploid (2*x*) and fertilized by a haploid male gamete (*x*), the resulting endosperm is triploid (2 + 1) and, therefore, genetically distinct from the diploid embryo (1 + 1). The endosperm of most angiosperms is triploid, with a 2:1 ratio of the maternal to the paternal genome, although exceptions have been found in some species (Williams and Friedman, 2002; Mizuochi *et al.*, 2009). Endosperm is particularly sensitive to ploidy unbalance (Köhler *et al.*, 2010). An unbalanced endosperm may lead to an unviable seed (Costa *et al.* 2004). Deviations from the ratio of two maternal (2m) to one paternal (1p) genome in the endosperm can cause endosperm failure (Köhler *et al.*, 2010). Increased contributions of maternal or paternal genomes inhibit proliferation of the endosperm or cause endosperm excess, respectively (Scott *et al.*, 1998).

*Dioscorea alata* breeding programmes were exclusively based on the creation of diploid varieties until 2006, although polyploidy has been acknowledged for a long time (Ramachandran, 1968; Abraham and Nair, 1991). The first polyploid hybrids were recently created by conventional hybridization, thanks to the discovery of the fertility of *D. alata* tetraploid varieties and to the development of an *in vitro* immature embryo rescue method (Arnau *et al.*, 2006; Abraham and Arnau, 2009). Intercytotype crosses between diploid females and tetraploid males revealed the phenomenon of endosperm incompatibility because they produce non-viable mature seeds with shrivelled endosperm. Obtaining tetraploid hybrids from intercytotype crosses suggested the ability of diploid progenitors to produce unreduced (2*n*) gametes, pollen or ovules. In the case of *D. alata*, triploid males are sterile because their flower buds remain unopened until drying off (Abraham and Nair, 1991), and the fertility of female flowers has never been demonstrated.

Flow cytometry is a fast and easy technique to determine ploidy levels in plants (Doležel *et al.*, 1994; Seker *et al.*, 2003) and has already been used to screen 2*x* × 2*x D. alata* progenies (Arnau *et al.*, 2011). Flow cytometry has also been used to determine endosperm ploidy in several species and to better understand the phenomenon of endosperm incompatibility in interspecific or intercytotype crosses (Pichot and Maataoui, 1997; Sliwinska *et al.*, 2005).

Because microsatellite markers are co-dominant and highly reproducible, they are suitable for the analysis of allele segregation in progenies (Ashley and Dow, 1994). Once maternal and paternal microsatellite genotypes are known, the different pathways for gamete production and unions that give rise to polyploid individuals can be compared for their likelihood. For example, the origin of 2*n* gametes (maternal or paternal) can be deduced from microsatellite analysis. The transmitted heterozygosity can also provide knowledge about the type of mechanisms involved in the non-reduction, i.e. the suppression of restitution of the first or the second division.

Implication of gametic non-reduction in the formation of polyploid individuals has never been demonstrated in *D. alata*. Furthermore, endosperm incompatibilities in this species have never been studied, in spite of their importance in intercytotype crosses. This knowledge is crucial for the optimization of the production of new polyploid (3*x* and 4*x*) cultivars.

The objective of the present study was to use flow cytometry and microsatellite markers to understand the origin of *D. alata* spontaneous triploids and tetraploids. Diploid genitors suspected of producing unreduced gametes were used. Intercytotype crosses between diploid, triploid and tetraploid genitors generated different types of progeny. Flow cytometry was used to measure embryo and endosperm ploidy. Microsatellite markers were genotyped on diploid parents and their detected polyploid offspring. This work made it possible to identify the origin of 2*n* gametes and endosperm, and provided knowledge about the formation of *D. alata* polyploids.

## MATERIALS AND METHODS

### Plant materials

The origin of the *Dioscorea alata* plant material used is given in Table 1.

**Table 1.** Origin of plant material

Plant	Type	Origin	Ploidy
‘5F’	Hybrid	Guadeloupe	2*x*
‘Kabusa’	Local cultivars	Caraîbes	2*x*
258F	Local cultivars	Madagascar	3*x*
148	Local cultivars	Vanuatu	4*x*

The first progeny of 300 seeds was obtained by crossing two diploid parents (2*n* = 2*x* = 40) (female ‘5F’ and male ‘Kabusa’). The diploid status of ‘5F’ and ‘Kabusa’ was ascertained by flow cytometry in Arnau *et al.* (2009). Both diploid genitors are suspected of producing unreduced gametes. Half of the seeds (*n* = 150) were sown, and fresh leaves of seedlings were analysed using flow cytometry. The other half of the seeds were desiccated 90 d after pollination and the endosperm was separated from the embryo. Flow cytometry was then performed on seedlings resulting from embryo culture, whereas a joint flow cytometry analysis was carried out on endosperm. Simple sequence repeat (SSR) analyses were only performed on the leaves of detected triploid seedlings.

The second progeny of 2000 seeds obtained by crossing the diploid female clone ‘5F’ and the tetraploid male clone 148 was used: (1) to study endosperm incompatibility in 2*x* × 4*x* crosses; and (2) to analyse events of non-gametic reduction in the female parent. One thousand seeds were sown to evaluate seed viability and to check the ploidy of emerging plantlets by flow cytometry. The remaining 1000 seeds were desiccated to allow a separate ploidy analysis on endosperm and embryos.

The gametic fertility of a triploid female clone (258F) was analysed by crossing it with the diploid male ‘Kabusa’. The fertility study of the male ‘Kabusa’ was done by staining the pollen with carmine. Obtaining tetraploids by this cross would be indicative of the formation of unreduced balanced gametes in triploid females. A total of 300 female flowers were fertilized by manual hybridizations, corresponding to 1800 potential seeds, given that a fruit can contain 1–6 seeds. The rates of fruit set and seed setting were recorded.

### Flow cytometry analysis

Flow cytometry analysis of leaves was performed as described by Arnau *et al.* (2009). The nuclear DNA content of samples was determined by comparison of the relative positions of the G_0–1_ peak of different internal references: 760a as the triploid reference, 639a as the diploid reference and 754a as the tetraploid reference. The ploidy of these three clones was determined by mitotic chromosome counts in Arnau *et al.* (2009). For endosperm analysis, the protocol described by Sliwinska *et al.* (2005) was used, with some adaptations. Endosperm was chopped up with a double-edged razor blade in 1 mL of nucleus isolation buffer [0·1 m Tris–HCl, 2·5 mm MgCl_2_·6H_2_O, 85 mm NaCl, 1 % (w/v) polyvinylpyrrolidone-PVP-10 and 0·1 % (v/v) Triton X-100 pH 7]. The suspension was filtered through a 30 µm pore filter. A 300 µL aliquot of filtrated endosperm solution, 200 µL of filtrated leaf solution of an internal standard and 400 µL of isolation buffer supplemented with propidium iodide (50 µg mL^−1^) and RNase A (50 µg mL^−1^) were then mixed in a tube. The suspensions were incubated for approx. 5 min at room temperature. After incubation, each sample was run on a flow cytometer. DNA quantities were measured using a FACScalibur laser flow cytometer (Beckton Dickinson, USA) with Cellquest Software.

The choice of the internal reference was made according to the expected offspring or endosperm ploidy. To detect non-expected seedlings (triploid in a diploid population or tetraploid in a triploid population), an appropriate internal reference [diploid (639a) or triploid (760a), respectively] was used and the results were interpreted as follows. The internal reference produces two fluorescence peaks: a major peak corresponding to 2*x* or 3*x* DNA quantities of the majority of leaf cells, and a replicated G_2_ minor peak corresponding to 4*x* or 6*x* DNA quantities from cells in mitotic interphase. When the fluorescence peak corresponding to the nuclei obtained from a given sampled individual was between these two reference peaks, the individual was assumed to be triploid with the diploid internal reference or tetraploid with the triploid internal reference if the sample peak is closer than the G_0–1_ peak. When no additional peak was observed between the two peaks of the internal reference, the individual was assumed to have the same ploidy level as the reference. The same principle is applied to measure expected hexaploid (6*x*) endosperm. The tetraploid (754a) internal reference was used and produced two fluorescence peaks. When the fluorescence peak corresponding to the nuclei obtained from a given sampled endosperm was equidistant from these two reference peaks, the endosperm was assumed to be 6*x*.

### Microsatellite amplification

Seventy-five SSRs developed from five different yam species – *D. rotundata*, *D. abyssinica*, *D. prahensilis*, *D. japonica* and *D. alata* (Misuki *et al.*, 2005; Tostain *et al.*, 2006; Andris *et al.*, 2010) – were used to determine the genotypes of the genitors. Only the six SSR markers that revealed polymorphism between the diploid parents ‘5F’ and ‘Kabusa’, without any common allele (Da2F10, Da1D08, mDaCIR8, mDaCIR60, mDaCIR61 and mDrCIR128), were selected. Primer sequences are given in Table 2. Da2F10, mDaCIR8, mDaCIR61 and mDrCIR128 show two alleles in the female progenitor ‘5F’ and two different alleles in the male progenitor ‘Kabusa’. Da1D08 and mDaCIR60 show heterozygosity in ‘5F’ and are homozygous in ‘Kabusa’ but with different alleles.

**Table 2.** Primer sequence of the six microsatellite markers using for study of the allelic contribution of the progenitor

Locus	Sequence F	Sequence R	Origin
Da2F10	TCAAGGATAAGAACTCCC	CAACGGCTAAACAGAAA	*D. alata*
Da1D08	GATGCTATGAACACAACTAAC	TTTGACAGTGAGAATGGA	*D. alata*
mDaCIR8	ACAGCAGCAAAATAACTG	TCTTTGCAGGAGAAGAGG	*D. alata*
mDaCIR60	CAAAGACCAGGGAATGTG	AGAATGCAGAGCATGGTG	*D. alata*
mDaCIR61	CTAACCCCTCCAAAGCTG	GGGCATTCACGTCTTTAT	*D. alata*
mDrCIR128	CCGTATTCCAAGCGATAA	AGCGTGAAAACCTGATAAAA	*D. rotundata*

The forward sequence (sequence F) and reverse sequence (sequence R) are given.

Amplification was performed in a total volume of 20 µL containing 0·05 U μL^−1^ of *Taq* polymerase, 2 µL of 10× buffer, 0·2 mm dNTP, 2 µmol of labelled or unlabelled primers, 2 mm MgCl_2_ and 10 ng of DNA. Forward primers were labelled with one of the following fluorophores: TET, NED, HEX or 6-FAM. The PCR conditions were as follows: 5 min of denaturation at 94 °C, followed by 30 cycles alternating 30 s of denaturation at 94 °C, 30 s of hybridization at annealing temperature, 35 s of extension at 72 °C, and ending with 5 min of final elongation at 72 °C. PCR was carried out using a PTC100 thermocycler (MJ Research). Electrophoregrams were obtained by migration of amplification products on an ABI PRISM-TM 3100 automatic sequencer (Applied Biosystems). Allelic profiles were determined using GeneMapper v3·7 software (Applied Biosystems). Parents and polyploid zygotes eventually found in 150 seeds (second seed lot) of the ‘5F’ × ‘Kabusa’ population were genotyped.

## RESULTS

### Triploids from the 2*x* × 2*x* cross: ‘5F’ × ‘Kabusa’

#### Frequency

Out of the 300 plantlets or embryos of the ‘5F’ × ‘Kabusa’ progeny, four were found to be 3*x* (1·3 %) and 296 were found to be 2*x* (98·7 %). The diploid internal reference 639a was used for flow cytometry.

**Fig. 1.**
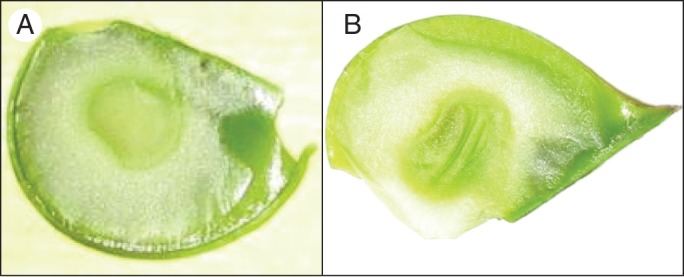
Photographs of a plump seed in (A) and a shrivelled seed in (B).

The aspect of all the sown seeds that produce seedlings (diploid and triploid) was normal (plump) (Fig. 1A). No clear morphological differences made it possible to differentiate *a priori* 3*x* seeds from 2*x* seeds.

#### Endosperm ploidy and gamete origin

**Fig. 2.**
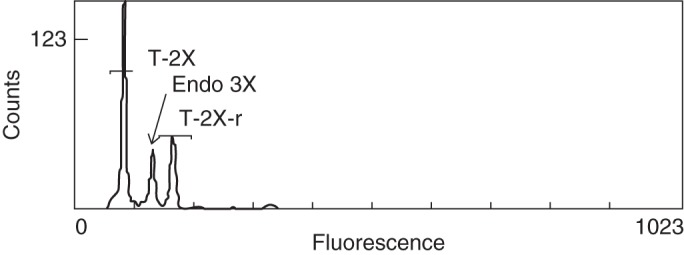
Flow cytometry analysis of detached endosperm from desiccated seeds from which triploid plants grew. Seeds were obtained on the ‘5F’ × ‘Kabusa’ cross, both 2*x* parents. The diploid internal reference (639a) produced two fluorescence peaks: T-2X, the major G_0–1_ peak corresponding to a 2*x* DNA quantity of the majority of leaf cells; and T-2X-r, the replicated G_2_ minor peak corresponding to the 4*x* DNA quantities of cells in mitotic interphase. The fluorescent peak corresponding to endosperm (Endo 3X) is equidistant to the two reference peaks and is triploid (3*x*).

Out of the 150 seed lot for which endosperm was detached, flow cytometry was possible on two seeds that then produced 3*x* plants, hereafter referred to as 5K1 and 5K5. The diploid internal reference 639a was used for flow cytometry. Endosperm peaks for the two seeds corresponded to a 3*x* state (Fig. 2). Two other seeds that also produced 3*x* embryos could not be analysed because of a shrivelled endosperm (Fig. 1B). These two 3*x* individuals were obtained by embryo rescue and are hereafter referred to as 5K11 and 5K34.

**Fig. 3.**
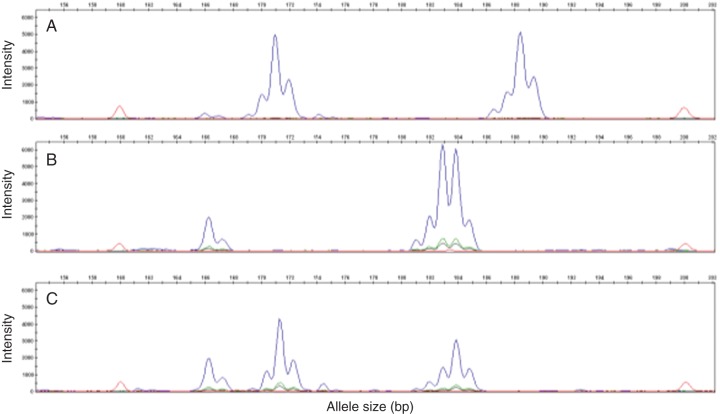
Phenomenon of non-reduction of the male gamete. Electrophoregram at locus CIR8 (A) for the diploid female progenitor ‘5F’, (B) for the diploid male progenitor ‘Kabusa’ and (C) for one triploid hybrid 5K34 with shrivelled endosperm. ‘5F’ have phenotype 171/188; ‘Kabusa’ have phenotype 166/184; 5K34 have phenotype **166**/171/**184** (bold indicates the paternal allelic contribution).

Parental (‘5F’ and ‘Kabusa’), 5K1, 5K5, 5K11 and 5K34 genotypes for the six microsatellite markers are given in Table 3. Microsatellite analysis conclusions on the origin of the 3*x* individuals were drawn as follows. When the 3*x* offspring carried the two alleles of the female (‘5F’) and only one allele from the male (‘Kabusa’), it was assumed that the female produced an unreduced ovule and the male a normal reduced pollen grain. When the two paternal alleles and only one maternal allele were transmitted, the unreduced gamete was assumed to be a pollen grain. In Fig. 3, the case of the 5K34 seedling is illustrated for locus mDaCIR8. The two male alleles (166 and 184 bp) are present in 5K34 with only one of the maternal alleles (171 bp). It can be postulated at this locus that 5K34 resulted from an unreduced pollen grain and a normal ovule. For other loci (mDaCIR61 and mDrCIR128), heterozygosity was not transmitted by the male or by the female.

**Table 3.** Parental and hybrid genotypes at six SSR markers

	Da2F10	Da1D08	mDaCIR8	mDaCIR60	mDaCIR61	mDrCIR128
‘5F’	126/132	303/313	171/188	146/157	200/217	286/310
‘Kabusa’	124/130	306	166/184	142	188/193	300/308
5K5	_	_	166/171	142/146/157	188/200/217	308/310
5K34	_	303/306	166/171/184	142/146	188/200	300/310
5K1	124/126/132	303/306/313	166/171	_	_	300/310
5K11	124/132	_	166/184/188	142/146	_	286/308

The female progenitor ‘5F’, the male progenitor ‘Kabusa’ and four of their offspring, 5K1, 5K5, 5K11 and 5K34, were analysed (out of a 300 progeny). Alleles are given in bp.

Identically, triploid individuals obtained from plump seeds, 5K5 and 5K1, received heterozygosity from ‘5F’ on loci Da2F10 and Da1D08. It can be hypothesized that they arise from a non-reduced ovule (Table 3). Triploids obtained from shrivelled seeds, 5K34 and 5K11, are assumed to come from non-reduced pollen grains.

They are not the same loci that transmit male or female heterozygosity. On average, the rate of transmitted heterozygosity is 50 % for 2*n* ovules (the case of 5K1 and 5K5) and approx. 22 % for 2*n* pollen (5K11 and 5K34).

### Offspring of the female 2*x* × male 4*x* cross: ‘5F’ × 148

Out of the 1000 sown seeds obtained by crossing the diploid female (‘5F’) and the tetraploid male (148), 18 seedlings germinated from seeds with a plump aspect. Flow cytometry showed that these seedlings were all tetraploids. The triploid internal reference was used.

**Fig. 4.**
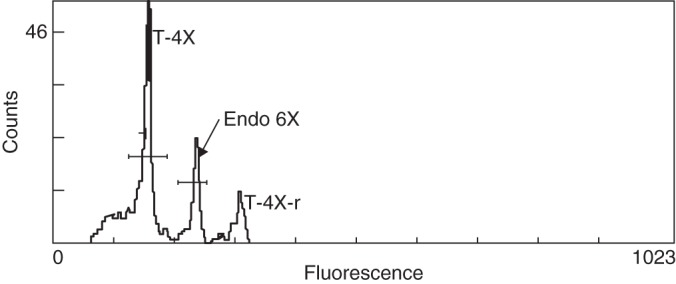
Flow cytometry analysis of detached endosperm from desiccated seeds from which tetraploid plants grew. Seeds were obtained on the ‘5F’ (2*x*) × 148 (4*x*) cross. The tetraploid internal reference (754a) produced two fluorescence peaks: T-4X, the major G_0–1_ peak corresponding to a 4*x* DNA quantity of the majority of leaf cells; and T-4X-r, the replicated G_2_ minor peak corresponding to the 8*x* DNA quantities of cells in mitotic interphase. The fluorescent peak corresponding to endosperm (Endo 6X) is equidistant to the two reference peaks and is hexaploid (6*x*).

Out of the 1000 dessicated seeds, 995 had a shrivelled aspect and five were plump. Flow cytometry was carried out on 100 of the 995 shrivelled detached endosperms, but only six had a sufficient signal. These six endosperms had a signal peak between the two reference peaks of the triploid internal reference ‘760a’ (3*x* and 6*x* for the G_2_ peak). They were therefore assumed to be 4*x*. The corresponding embryos of these 100 shrivelled seeds were positioned as triploids using flow cytometry. Flow cytometry analysis showed that embryos of the five plump seeds were tetraploid. The endosperm peak of these five seeds was located at a 6*x* position (Fig. 4) when the tetraploid internal reference was used.

### Fertility of triploid females: 258F × ‘Kabusa’

Results of fertility study on the male ‘Kabusa’ showed that the pollen viability is high (80 %). A very small number of seeds was obtained by crossing the triploid female 258F with the diploid male ‘Kabusa’ (only 18 out of an optimal number of 1800 seeds). Half (nine) of these seeds lacked an embryo. The other half contained embryos but none of them evolved into a viable seedling, even if development was initiated in two of them.

## DISCUSSION

This work is the first study reported in Dioscoreacea where seeds were desiccated to allow separate ploidy analysis on endosperm and embryos. However, it appeared that although endosperm analysis is efficient for plump seeds, it is not easy to apply to shrivelled seeds. Because polyploids were obtained at a low rate, this could lead to doubts about the sample size of the progeny used to obtain accurate estimates of non-reduced gamete formation. Our results should be considered more as qualitative rather than accurate estimates.

In this study, 2*x* × 2*x* crosses gave 1·3 % of 3*x* individuals with a contrasted endosperm aspect. The flow cytometry of joint embryos and endosperms, seed aspects and microsatellite analysis together led to evidence that both male and female clones produced 2*n* gametes that mated with *x* gametes to produce viable 3*x* plants. When the unreduced gamete was an ovule, endosperm ploidy was 3*x* and the seed was plump. When the unreduced gamete was a pollen grain, endosperm was shrivelled and its ploidy level could not be determined.

In the same manner, 4*x* individuals were obtained in the 2*x* × 4*x* cross (1·15 %). These seeds are easily identified by the plump aspect of their seeds, corresponding to the expected 6*x* endosperm if the diploid female produced a 2*n* ovule and if the endosperm originated from the fusion of the two 2*n* cells of the embryo sac (2*x* + 2*x*) with the normal 2*x* male gamete produced by the 4*x* male. For the other seeds, embryos were 3*x* and the endosperm was shrivelled, consistent with the union of a normal reduced ovule (*x*) from the diploid female and a reduced pollen grain from the 4*x* male.

This suggests that the production of 2*n* gametes is at the origin of *D. alata* polyploids, as is the case for potatoes (Iwanaga and Peloquin, 1982; Carputo *et al.*, 2000) and many other species (for a review, see Ramsey and Schemske, 1998).

Other phenomena such as polyspermy (fertilization of an egg by two sperm nuclei) and endospermal polyembryony (formation of embryos from endosperm cells) have been suggested as possible mechanisms that could lead to the production of triploids in other species. Polyspermy is not considered as a likely mechanism in the formation of polyploids (Harlan and DeWet, 1998) but has been demonstrated in the genus *Juglans* (Navashin and Finn, 1951). In *D. alata*, neither polyspermy nor polyembryony can explain the microsatellite profiles obtained on the triploids for the six loci tested. Under the polyspermy hypothesis, regardless of the paternal genotype, individuals would carry only one of the paternal alleles at all loci because the two sperm nuclei are identical by mitosis (Borg *et al.*, 2009). For some loci, paternal heterozygosity in triploids was observed and makes it possible to eliminate polyspermy. Endospermal polyembryony, which consists of the formation of embryos from endosperm cells, was observed in the genera *Bracharia* (Muniyamma, 1977), *Beta* (Yarmolyuk *et al.*, 1990) and *Citrus* (Gmitter *et al.*, 1990). These examples are rare and weakly substantiated (Batygina and Vinogradova, 2007). In this case, regardless of the maternal genotype, individuals would carry only one maternal allele at all loci since the two polar nuclei are identical by mitosis. Because maternal heterozygosity was observed in our *D. alata* triploid individuals at some loci, polyembryony may also be eliminated.

All these elements make it possible to conclude that triploid *D. alata* originate from 2*n* gamete formation. Two basic types of meiotic restitution mechanisms that lead to 2*n* gamete formation have been reported: FDR and SDR (Mok and Peloquin, 1975; Park *et al.*, 2007). These two cytological events do not transmit the same parental heterozygosity to their progeny. FDR mechanisms lead to 2*n* gametes that contain non-sister chromatids between the centromere and the first crossover. Consequently, all loci between the centromere and the first crossover that were heterozygous in the diploid parent will be heterozygous in the 2*n* gametes. Half of those beyond the crossover will be heterozygous in the gametes. SDR mechanisms lead to 2*n* gametes that contain sister chromatids between the centromere and the first crossover. All loci between the centromere and the first crossover that were heterozygous in the diploid parent will be homozygous in these 2*n* gametes, whereas those beyond the crossover will be heterozygous (Peloquin *et al.*, 2008). As a result, heterozygosity transmission by FDR varies from 100 to 50 %, and transmission by SDR varies from 0 to 100 % and depends on the position of markers in relation to the centromere (Park *et al.*, 2007).

Unreduced gametes were observed in the two sexes with a similar low probability (<2 %). Microsatellite markers were used to detect non-gametic reduction in males and females by studying heterozygosity transmission. Our preliminary results would indicate that unreduced ovules could transmit more heterozygosity than unreduced pollen. However, our study clearly lacks the statistical power to draw a final conclusion: a greater number of 3*x* individuals should be genotyped on a greater number of SSRs. Further analysis of PMCs (pollen mother cells) could also help to determine the type of 2*n* gametes in males.

Unreduced gamete formation makes it possible to overcome post-zygotic barriers that occurred in interploid crosses due to endosperm abortion (Peloquin *et al.*, 1999). Most of the expected endosperm ploidy levels were observed in the different crosses [♀2*x* (*n*) × ♂4*x*, ♀2*x* (2*n*) × ♂4*x*] with the remarkable exception of the 2*x* × 2*x* cross with the formation of 2*n* ovules. In this latter case, the 3*x* individual resulted from plump seeds with a 3*x* endosperm. Histochemical analysis in *Dioscorea nipponica* by Torshilova *et al.* (2003) suggests that the embryo sac of Dioscoreacea would be of the monosporic octonuclear type, Polygonum, which leads to a 3*x* endosperm with a 2:1 ratio in a usual diploid cross. Therefore, the embryo sac of a diploid female that produces a 2*n* ovule is expected to contain two 2*n* polar nuclei. After fertilization by a normal male gamete, these two polar nuclei should normally generate a non-viable 5*x* endosperm with a 4:1 ratio. Since the observed endosperm ploidy was 3*x*, this suggests that only one of the two polar nuclei could have been fertilized. This phenomenon has been observed in other species such as in *Triticum aestivum* (You and Jensen, 1985). For triploid individuals resulting from a union between unreduced pollen and a reduced ovule, endosperms were shrivelled. Their expected endosperm ratio is 2:2, which, unfortunately, could not be measured in our experiment. This suggests that this type of gametic combination should not lead to the production of viable seeds as a consequence of endosperm dysfunction.

No triploid seedlings were obtained from the 1000 seeds from the intercytotype cross, 2*x* female × 4*x* male. Embryos were 3*x* but their endosperm were found to be 4*x*, consistent with the expected 2:2 maternal to paternal genome ratio, confirming that in *D. alata*, unbalanced endosperm leads to shrivelled seeds and unviable seeds. Since *in vitro* embryo rescue on such crosses gives rise to 3*x* hybrids, this scenario is confirmed.

**Fig. 5.**
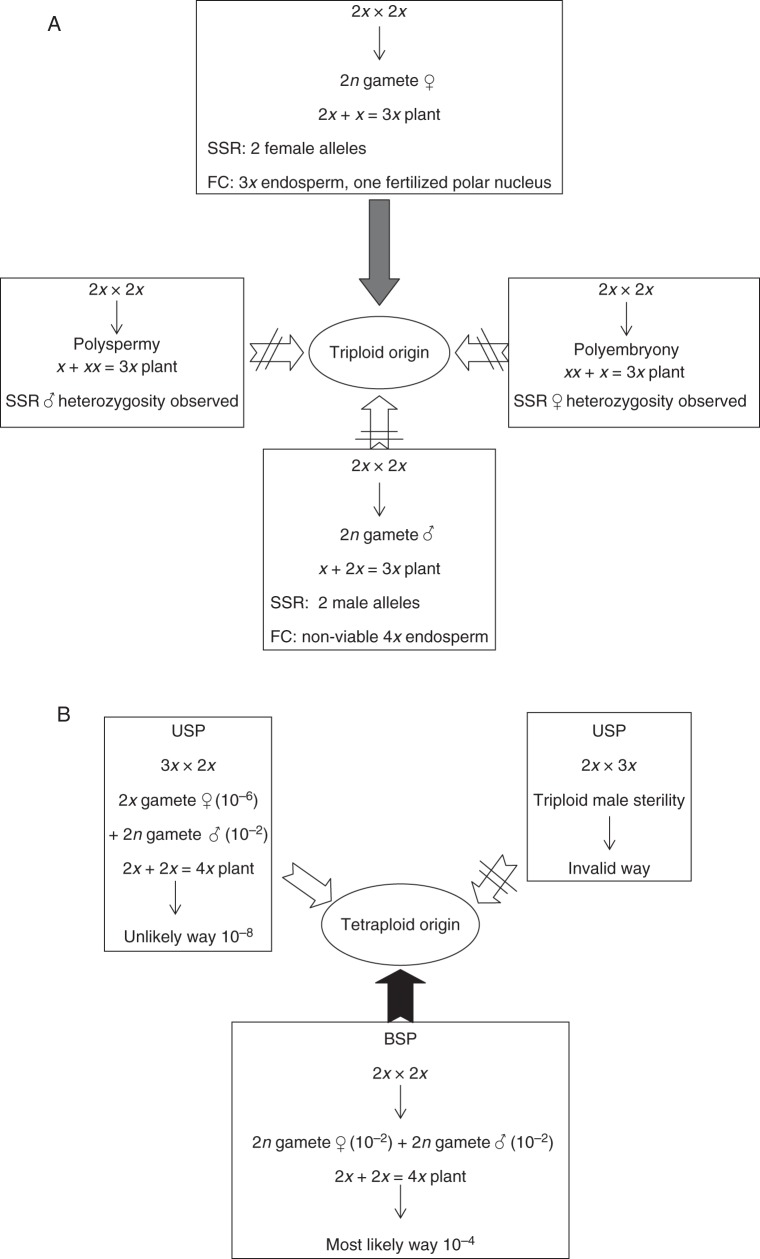
Scheme representing the different pathways for the origins of *D. alata* polyploids. Triploid origin in (A) and tetraploid origin in (B). SSR, microsatellite analysis; FC, flow cytometry analysis; // indicates an invalid route.

In brief, since plump seeds from 2*n* ovules × *n* pollen have a better chance of germinating in the wild than shrivelled seeds from an *n* ovule × 2*n* pollen, the likely spontaneous triploid formation in *D. alata* is the cross of a diploid maternal gamete with a haploid paternal gamete (Fig. 5A).

Since 3*x* are highly infertile, the existence of fertile 4*x* plants remains to be documented. Theoretically, the two different ways that could have led to the production of the first tetraploids are USP (triploid bridge) and BSP (fusion of two unreduced gametes). Obtaining tetraploids via BSP has been demonstrated in several autopolyploid species such as red clover (Parrott *et al.*, 1985) and *Dactylis glomerata* (Bretagnolle and Lumaret, 1995). In the 2*x* × 2*x* progeny, no tetraploid was obtained by BSP on the 300 seeds examined. With an observed rate of 2*n* gamete formation of 1·3 %, the theoretical chance of obtaining a tetraploid would have been 1/2500. If this scenario could be verified by examining a much larger descent than in this study, it could be considered as much more likely than the triploid bridge for *D. alata* (Fig. 5B). Furthermore, it would normally not have a problem of seed development with the BSP scenario because a normal 6*x* endosperm with a 2:1 (4:2) ratio is expected.

No tetraploid seedlings were obtained by triploid bridges on a descent of 2000 seeds (i.e. a cross between triploids and diploids to obtain tetraploids). Our results confirm a general picture of a very high sterility rate of triploid females, as reported by Abraham and Nair (1991). These authors concluded that triploid females are sterile based on a study carried out on 27 triploid females crossed with a male diploid (50–130 pollinations).

In triploids, segregation of chromosomes at meiosis is complex because of the trivalent formation at metaphase I, which would lead to a majority of aneuploid gametes after the second meiotic division. The probability of obtaining viable euploid gametes (balanced) depends on the *x* chromosome number. Given that each chromosome has a 50 % chance of migrating to one of the cell's poles at anaphase I, the frequency of obtaining haploids, diploids and triploids is 1/2^*x*^, 1/2^*x*^ + 1/2^*x*^ and 1/2^*x*^ × 3, respectively. The chromosome base number of most species for which the triploid bridge has been demonstrated is quite low. *Cucumis sativus*, in which two tetraploids were obtained by USP on 545 seeds, has *x* = 7 pairs of chromosomes (Diao *et al.*, 2009).

It has been demonstrated that non-reduction gametic phenomena can increase the frequency of obtaining diploid and triploid gametes in triploids by SDR and FDR, respectively (Husband, 2004). This is the case for *C. angustifolium* (*x* = 18) where the high rate of unreduced gametes made it possible to obtain tetraploids. The rate of 2*n* gametes observed in *C. angustifolium* species is ten times higher than that reported by Ramsey and Schemske (1998), based largely on crop plants. Moreover, in this species that has an Oenothera-type monosporic embryo sac, no endosperm incompatibility phenomenon was observed in intercytotype crosses (Husband, 2004). In *D. alata*, whose chromosome base number is *x* = 20 (Arnau *et al.*, 2009), viable haploid, diploid and triploid gametes are predicted at frequencies of 1/2^20^, 1/2^20^ + 1/2^20^ and 1/2^20^ × 3, respectively, corresponding to very low rates for obtaining viable seedlings. Screening of triploid females in other collections would make it possible to check if USP (triploid bridge) is a possible formation mechanism of tetraploids. However, in the CIRAD *D. alata* germplasm collection, no gametic non-reduction has yet been observed for triploid females (unpublished data) via USP checking. In *D. alata*, in addition to a very low theoretical frequency for obtaining triploid balanced gametes (three out of 1 million), it would be assumed that endosperm incompatibility would lead to a heptaploid endosperm with a ratio of 6:1 (or tetraploid with a 3:1 ratio if only one polar nucleus is fertilized) and a likely non-viability for the seeds.

Moreover, our results revealed that in *D. alata*, as in other species (Burton and Husband, 2001; Hardy *et al.*, 2001), tetraploids can be obtained by intercytotype crosses (2*x* × 4*x*) via gametic non-reduction in the diploid female progenitor. Thus, the gene flows between the diploid and tetraploid compartments may have contributed to enhancing diversity at the tetraploid level. Reciprocal crosses (4*x* × 2*x*), although not carried out in this study, could produce tetraploids via gametic non-reduction in the male diploid progenitor. Once the tetraploid pool is established, mating could start between 4*x* individuals and lead to the recombination of diversity at this ploidy level.

### Conclusions

The major conclusion of this study is that polyploids in *D. alata* might have appeared as a result of 2*n* gamete formation. The most likely origin of spontaneous triploids would be the union of an unreduced egg and a reduced pollen grain with normal 3*x* endosperm, whose formation is still unknown. Crossing a diploid female with a tetraploid male is a possible way to obtain triploids in *D. alata* but requires the use of embryo rescue, excluding the possibility that this mechanism could have contributed to enhancing the diversity of the wild triploid pool. Although tetraploids were not obtained in this experiment, probably since the sample of seeds germinated was too small, we could reasonably assume that their most likely primary origin in the wild would be BSP via the mating of two unreduced gametes produced by diploids. Gene flows between diploid and tetraploid compartments by intercytotype crosses (2*x* × 4*x*) may have further contributed to broadening the allelic diversity at the tetraploid level, while recombination at the 4*x* level created new gene combinations. Screening of *D. alata* collections worldwide could make it possible to identify diploid progenitors with high 2*n* gamete production and to use them to enlarge the genetic diversity in the cultivated polyploid compartment. Knowledge of the origin of 2*n* gametes, SDR or FDR, will make it possible to increase the transmission pathway of the parental heterozygosity rate.
